# Genomic Comparisons and Phenotypic Diversity of *Dickeya zeae* Strains Causing Bacterial Soft Rot of Banana in China

**DOI:** 10.3389/fpls.2022.822829

**Published:** 2022-02-09

**Authors:** Jingxin Zhang, Mohammad Arif, Huifang Shen, Dayuan Sun, Xiaoming Pu, John Hu, Birun Lin, Qiyun Yang

**Affiliations:** ^1^Key Laboratory of New Technique for Plant Protection in Guangdong, Institute of Plant Protection, Guangdong Academy of Agricultural Sciences, Guangzhou, China; ^2^Department of Plant and Environmental Protection Sciences, College of Tropical Agriculture and Human Resources, University of Hawai’i at Mānoa, Honolulu, HI, United States

**Keywords:** *Dickeya zeae*, bacterial soft rot of banana, genomic comparisons, phenotypic diversity, pathogenicity

## Abstract

Bacterial soft rot of banana, caused by *Dickeya zeae*, is spreading rapidly in important banana growing areas in China and seriously threatens banana production. In this study, we sequenced the high-quality complete genomes of three typical banana strains, MS1 (size: 4,831,702-bp; genome coverages: 538x), MS_2014 (size: 4,740,000-bp; genome coverages: 586x) and MS_2018 (size: 4,787,201-bp; genome coverages: 583x), isolated in 2009, 2014, and 2018, respectively. To determine their genomic and phenotypic diversity with respect to their hosts of origin, they were compared with other *D*. *zeae* strains, including another representative banana strain MS2 from China. The sequenced strains were similar in utilization of carbon source and chemical substrates, and general genomic features of GC content, and tRNA and rRNA regions. They were also conserved in most virulence determinants, including gene-encoding secretion systems, plant cell wall degrading enzymes, and exopolysaccharides. We further explored their genomic diversity in the predicted genomic islands (GIs). These GIs were rich in integrases and transposases, where some genomic dissimilarity was observed in the flagellar gene cluster and several secondary metabolite gene clusters. Different constituents of core biosynthetic modules were found within the bacteriocin and aryl polyene (APE) pigment gene clusters, and the strains from banana showed different phenotypes with respect to antibiosis effects and colony pigmentation. Additionally, clustered regularly interspaced short palindromic repeat (CRISPR) and prophage elements, such as type I-F and III-A CRISPR arrays and an intact prophage of MS1-P5, contributed to bacterial diversity. Phylogenetic tree analysis and genome-genome nucleotide comparison confirmed the genomic divergence among the strains isolated from banana. Considering these characteristics, MS2 and MS_2014 probably diverged later than MS1, while MS_2018 was different and more similar to foreign strains isolated from other hosts in several characteristics. Strain MS_2018 caused severe symptoms on banana varieties previously considered moderately resistant or moderately susceptible, including varieties of Cavendish (*Musa* AAA) and Plantain (*Musa* ABB). Our study of genomic and phenotypic diversity raises public attention to the risk of spreading new pathogenic variants within banana growing regions and supports development of predictive strategies for disease control.

## Introduction

Banana (*Musa* spp.) is an important fruit in tropical and subtropical regions and an essential food crop in some developing countries. In 2019, the total harvested area worldwide was 5,158,582 ha, with a production of 116,781,658 tonnes. The top five countries in area planted were: India, Brazil, China, the United Republic of Tanzania, and Rwanda^1^. The long-term cultivation of a single banana cultivar and the changes in cultivation patterns and climate conditions have driven pathogen variation, resulting in the emergence of new banana diseases. In 2009, we first reported bacterial soft rot of banana caused by *Dickeya* sp. in mainland China (Lin et al., 2010). *Dickeya* spp. are Gram-negative and necrotrophic bacteria in family Pectobacteriaceae, and the causative agents of bacterial soft rot in a wide range of hosts. This banana disease spread rapidly to most banana-growing regions, including Guangdong, Guangxi, Yunnan, Fujian, and Hainan Provinces in China, causing significant losses to the susceptible variety *Musa* ABB Pisang Awak (Zhang et al., 2014; Fan et al., 2016; Du et al., 2019). *Dickeya paradisiaca* was previously discovered infecting banana (Dickey and Victoria, 1980; Samson et al., 2005), and we first reported *D*. *zeae* as the causative agent in China (Zhang et al., 2014).

*Dickeya zeae* has been found associated with various hosts, including rice, maize, banana, pineapple, taro, and Clivia (Hussain et al., 2008; Sueno et al., 2014; Zhang et al., 2014, 2020; Hu et al., 2018; Boluk et al., 2021). Among these pathogens isolated from assorted plants, different sequence variants (sequevars) were found (Parkinson et al., 2009). The strains from rice, along with some closely related strains, were proposed as a genomospecies, *D*. *oryzae* (Wang et al., 2020), but they were not distinctly separated from other *D*. *zeae* strains. In the pathogenesis among *Dickeya* pathogens, some typical virulence factors are mainly involved, such as: the type I secretion system (T1SS) and metalloproteinase (Hommais et al., 2008), T2SS and plant cell wall degrading enzymes (PCWDEs) (Lindeberg et al., 1998), T3SS and effector proteins (Yang et al., 2008), flagella and flagella-mediated motility (Antúnez-Lamas et al., 2009), T6SS and Rhs effectors (Russell et al., 2014), quorum sensing signal molecules AHL and Vfm (Nasser et al., 1998, 2013), second messenger molecule c-di-GMP (Chen et al., 2016), and zeamines limited within strains isolated from rice (Wu et al., 2010). *Dickeya zeae* strains isolated from rice, banana, and Clivia showed phenotypic divergence in their production of extracellular enzymes, extracellular polysaccharides, and antibiotic phytotoxin (Hu et al., 2018). However, few studies have been performed on the variation of pathogenicity or other phenotypes in *Dickeya* strains isolated from the same host and their interactions with host plants. Thus, the genetic mechanism underlying variations in virulence among different *Dickeya* strains and their interactions with host plants needs more attention. Genomic comparison have been performed in different *Dickeya* species, including some relatively newly determined species like *D*. *solani* (Pédron et al., 2014; Golanowska et al., 2018; Motyka-Pomagruk et al., 2020), *D*. *fangzhongdai* (Zhang et al., 2018; Alič et al., 2019), and *D*. *aquatica* (Duprey et al., 2019). Different with the high homogeneity shared by *D*. *solani* strains (Golanowska et al., 2018; Motyka-Pomagruk et al., 2020), we previously find high genomic heterogeneity occurred among different *D*. *zeae* strains isolated from different hosts. In this study, we isolated three typical *D*. *zeae* strains from banana in Nansha, Guangzhou, China, in three different years. To understand the genomic virulence factors involved in their differentiation and possible phenotypic diversity with respect to their hosts of origin, we performed genome-wide comparisons and found that these strains varied in phenotypes, including in colony morphology, antimicrobial activity, and virulence.

## Materials and Methods

### Bacterial Strains and Culture Media

Typical *D*. *zeae* strains isolated from banana, MS1, MS_2014 and MS_2018, were isolated from diseased banana in 2009, 2014, and 2018, respectively, when this bacterial disease accidentally outbroke and caused severe losses. All these three stains were isolated from Nansha, Guangzhou, China and stored at –80°C at the Key Laboratory of New Techniques for Plant Protection in Guangdong, China. For subculture, bacteria were grown on Luria-Bertani (LB) agar, and a single colony was isolated and cultured in LB broth in an incubator shaker at 100 rpm and 30°C.

### BIOLOG Analysis

Strains MS1, MS_2014, and MS_2018 were characterized using a GEN III MicroStation (BIOLOG, Hayward, CA, United States) to explore the biochemical properties of the strains. GEN III MicroStation provides a test to profile and identify a broad range of Gram-negative and Gram-positive bacteria. A 150-μL bacterial suspension (10^8^ CFU/mL) was added to each well of a GEN III microplate (BIOLOG) containing 94 substrates, including: 71 carbon source utilization assays and 23 chemical sensitivity assays. The MicroPlates were incubated for 14 h to form phenotypic fingerprint through the reduction of the tetrazolium redox dye caused by increased respiration. The phenotypic pattern in the GEN III MicroPlate was compared to the database information using the BIOLOG automated microbial analysis system software (GEN_ III_v2.8.0.15G).

### Genomic DNA Extraction and Genome Sequencing

Bacteria were grown to 10^8^ CFU/mL in LB broth and total DNA was extracted from each 2-mL bacterial suspension using a TIANamp Bacterial DNA Kit (Tiangen Biotech, Beijing, China) according to the manufacturer’s directions. Genomic DNAs were fragmented with G-tubes (Covaris, Woburn, MA, United States) and end-repaired. Repaired DNAs were selected by a Blue Pippin system to construct SMRTbell DNA libraries with fragment sizes of >10 kb according to the manufacturer’s specification (PacBio, Menlo Park, CA, United States). Library quality was determined using a Qubit 2.0 Fluorometer (Life Technologies, CA, United States), and the average fragment size estimated on a Bioanalyzer 2100 (Agilent, Santa Clara, CA, United States). At Genedenovo (Guangzhou, China), Pacific Biosciences Sequel (PacBio, Menlo Park, CA, United States) was used for SMRT sequencing according to standard protocols. Continuous long reads were attained from SMRT sequencing runs and were used for *de novo* assembly using Falcon (version 0.3.0) (Chin et al., 2016). Illumina sequencing was applied to correct the assemblies from PacBio. Genomic DNA was sonicated randomly, and the NEBNext^®^ MLtra™ DNA Library Prep Kit for Illumina (NEB, Ipswich, MA, United States) used for end-repair, A-tail, and adaptor ligation treatments. DNA fragments with lengths of 300–400 bp, enriched by PCR, were purified using the AMPure XP system (Beckman Coulter, Brea, CA, United States). The constructed libraries were analyzed for size distribution using a 2100 Bioanalyzer (Agilent, Santa Clara, CA, United States) and quantified using real-time qPCR. Genome sequencing was performed on an Illumina NovaSeq 6000 sequencer using paired-end technology (PE 150). Raw data from the Illumina platform were filtered using FASTP (version 0.20.0) (Chen et al., 2018) with the following filtering parameters: ≥10% unidentified nucleotides (N); ≥50% bases having phred quality scores ≤ 20; reads aligned to the barcode adapter. Filtered clean reads were aligned to the assemblies of PacBio sequencing. Inconsistencies between PacBio assemblies and Illumina reads were searched by Pilon (Walker et al., 2014), and the PacBio sequences were corrected and improved.

### Genome Annotation of *Dickeya zeae* Strains Isolated From Banana

The circular genomes were annotated using the Prokaryotic Genome Annotation Pipeline (PGAP) of NCBI (Tatusova et al., 2016). Repetitive elements, noncoding RNAs, and tRNAs were searched by repeatMasker (Chen, 2004), rRNAmmer (Lagesen et al., 2007), and tNRAscan (Lowe and Eddy, 1997), respectively. The genes predicted by PGAP were further annotated through similarity searches against different bioinformation databases including: the National Center for Biotechnology Information (NCBI) nonredundant protein (Nr) database, UniProt/Swiss-Prot, Kyoto Encyclopedia of Genes and Genomes (KEGG), Gene ontology (GO), Clusters of orthologous groups of proteins (COG), and Protein families (Pfam) databases. Clustered regularly interspaced short palindromic repeats (CRISPRs), gene clusters of polyketides (PKs) and nonribosomal peptides (NRPs), and putative prophage sequences were searched using CRISPRFinder (Grissa et al., 2007), antiSMASH 4.0 (Blin et al., 2017), and PHASTER (Arndt et al., 2016), respectively.

### Phylogenetic Analysis of *Dickeya zeae* Strains

Orthologous groups present as a single copy in genomes of 15 analyzed *D*. *zeae* strains (Table 1) were retrieved and their concatenated sequences used for phylogenetic analysis using MEGA 11 and the maximum likelihood method bootstrapped with 1,000 replications. The compared genome included a representative strain MS2 that is also isolated from banana in Nansha, Guangzhou, China in 2014. Average nucleotide identity (ANI) analysis was conducted for pair-wise genome comparison using the MUMmer algorithm (ANIm) on JSpecies Web Server (Richter et al., 2016). Additionally, *in silico* DNA-DNA hybridization (isDDH) values were calculated using the Genome-to-Genome Distance Calculator (GGDC) with formula 2 and BLAST+ alignment (Meier-Kolthoff et al., 2013). The ANI and isDDH data were presented in a color-coded heatmap by DISPLAYR.^2^

**TABLE 1:** Genomes of *Dickeya zeae* strains used for phylogenetic analysis and genomic comparison.

Strain name	NCBI accession number	Country	Host/Source	Contigs (assembly level)	Genome size (Mb)	GC%	CDS
MS1	NZ_CP053012	China	Banana	1	4.83	53.3	4122
MS2	NZ_CP025799	China	Banana	1	4.74	53.4	4068
MS_2014	NZ_CP053013	China	Banana	1	4.74	53.5	4035
MS_2018	NZ_CP053014	China	Banana	1	4.79	53.3	4084
MK19	NZ_CM001985	Scotland	River water	4	4.67	53.6	4071
NCPPB 2538*^T^*	NZ_CM001977	United States	Maize	7	4.56	53.6	3925
NCPPB 3532	NZ_CM001858	Australia	Potato	1	4.56	53.6	3947
CE1	NZ_CP033622	China	Canna	1	4.71	53.6	4037
Ech586	NC_013592.1	United States	Philodendron	1	4.82	53.6	4100
EC1	NZ_CP006929	China	Rice	1	4.53	53.4	3822
ZJU1202	AJVN01	China	Rice	188	4.59	53.3	3920
DZ2Q	APMV01	Italian	Rice	26	4.65	53.2	3934
EC2	NZ_CP031515	China	Rice	1	4.58	53.3	3881
NCPPB 3531	NZ_CM001980	Australia	Potato	2	4.63	53.7	3977
CSL RW192	NZ_CM001972	England	River water	4	4.70	53.4	4091

*^T^Indicates type strains.*

### Genomic Comparison of *Dickeya zeae* Strains From Banana or Other Hosts

The Blastatlas tool at the CGView server (Circular Genome Viewer)^3^ (Stothard et al., 2019) was applied for visualization of genomic comparison, and genome-to-genome comparisons were conducted using the tBlastX method with thresholds based on the e-value (1 × 10^–5^) and identity of full-length amino acids (80%). To further identify the common and specific genes among the 15 *D*. *zeae* strains (Table 1) and determine their orthologous relationships, we conducted BLAST searches of coding proteins on orthoMCL (Li et al., 2003) on the basis of the p-value cutoff (1 × 10^–5^) and identity cutoff (80%).

### Pathogenicity Test

Bacteria were grown to a concentration of 10^8^ CFU/mL in LB broth and diluted to 10^7^ CFU/mL with sterile water. A 400-μL bacterial dilution of each tested strain was injected into the basal stem of each banana seedling. Six different banana varieties were used for the pathogenicity test, including four varieties of *Musa* ABB Pisang Awak (Guangfen-1, Qingfen, Pinguofen, Fenza), one variety of *Musa* AAA Cavendish, and one variety of *Musa* ABB Plantain. Fifteen seedlings of each variety of were used for test, the tests were performed in triplicate. Banana seedlings inoculated with sterile water served as negative controls. The inoculated plants were placed in a greenhouse at 30°C and 90% relative humidity. After 7 days, the severity of banana soft rot symptoms was evaluated on a rating scale of 0–4: 0 = a healthy plant; 1 = that symptoms first appeared on the heart leaf; 2 = that symptoms appeared on the whole heart leaf, the leaves wilted, and symptoms first appeared on pseudostems; 3 = that the whole heart leaf was wilted, the upper leaves were yellowing, and the pseudostems were extremely soft; 4 = that the whole plant was wilting or dead. Disease indexes were calculated according to the formula: *Disease index* = ∑ (*number of diseased plants grouped into each rating value* × *representative rating value*)/(*the number of inoculated plants* × 4). Slides of potato tubers were used to evaluate the ability of the pathogen strains to induce soft rot symptoms (Joshi et al., 2015). Slides were externally disinfected by soaking in 0.7% sodium hypochlorite for 10 min, rinsing three times with sterile water, and air-drying at room temperature. *Dickeya zeae* strains isolated from banana were grown overnight in LB broth at 32°C with continuous shaking and diluted to 1 × 10^8^ cfu/mL in sterile water. Two microliters of the bacterial suspensions were spot inoculated onto the center of each slide using a sterile tip and the slides incubated for 24 h at 30°C and 90% relative humidity. Tests of each strain were performed in four repetitions. The area of disease spots was determined using ImageJ software.

Analyses of mean values, standard deviation values, and one-way ANOVA (p < 0.05) were calculated using the GraphPad Prism 6 (GraphPad Software, San Diego, CA, United States). Multiple comparisons of mean value differences were calculated with the Tukey’s test.

## Results

### Similar Substrate Use of *Dickeya zeae* Strains Isolated From Banana

**FIGURE 1:**
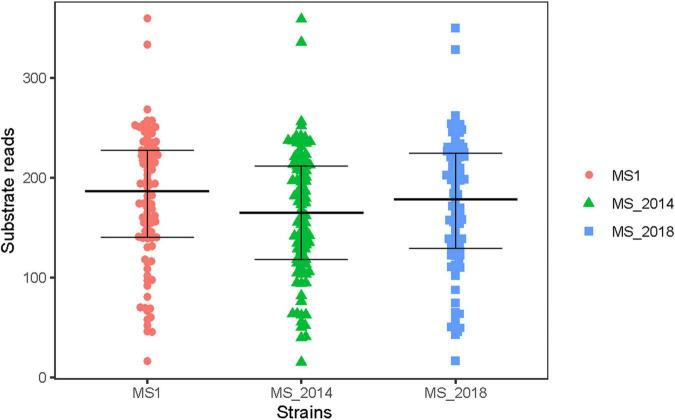
Biochemical characteristics of *Dickeya zeae* strains from banana using different substrates in BIOLOG analysis. In the BIOLOG assay, the tetrazolium redox dye in the cells induces reduction reaction in the increased respiration of bacteria, and produces purple color. Negative wells remain colorless. The beeswarm presents the original color densities in wells of the utilization assays of 94 substrates, and the bars from below to up indicate the lower quartile, median and upper quartile, respectively.

Compared to the BIOLOG databases, strains isolated from banana were confirmed to be within the *Dickeya* genus. They all were positive for the use of: D-raffinose, D-melibiose, α-D-lactose, D-mannitol, and myo-inositol, as was the case for the *D*. *zeae* species described by Samson et al. (2005). We found that only five substrates were distinct among the tested strains: *N*-acetyl-D-galactosamine, *N*-acetyl neuraminic acid, quinic acid, γ-amino-butyric acid, and α-hydroxy-butyric acid. These five substrates were carbon source, indicating the tested strains were similar in the use of most carbon source substrates and all of assayed chemical compounds. The beeswarm figure presenting the BIOLOG data also indicated that their use of most of analyzed substrates was similar (Figure 1).

### General Genomic Features of Strains From Banana

The complete genomes of strains MS1, MS_2014 and MS_2018 were 4,831, 702-, 4,740, 000-, and 4,787,201-bp long, respectively, and the genome coverages on each scaffold were 538.07×, 586.24×, and 583.35×, respectively (Table 2), indicating genomic assemblies of high quality. The three genomes had minor differences in GC content, numbers of predicted genes, and repetitive elements, and each had 75 tRNA and 7 rRNA regions in common.

**TABLE 2:** General genomic information for *D*. *zeae* strains isolated from banana.

Strains	MS1	MS2	MS_2014	MS_2018
Year of isolation	2009	2014	2014	2018
Location	Nansha, Guangzhou, China	Nansha, Guangzhou, China	Nansha, Guangzhou, China	Nansha, Guangzhou, China
Read bases from Pacbio (bp)	1,312,075,251	/	1,278,340,889	1,441,123,715
Read bases from Illumina (bp)	1,287,702,796	/	1,500,433,260	1,351,493,959
Genome size (bp)	4,831,702	4,740,052	4,740,000	4,787,201
Genome coverage	538.07x	104.00x	586.24x	583.35x
Predicted repetitive elements	207	204	204	223
Predicted rRNA regions	7	7	7	7
Predicted tRNAs	75	75	75	75

### Genomic Divergence Found Within Strains From Banana

**FIGURE 2:**
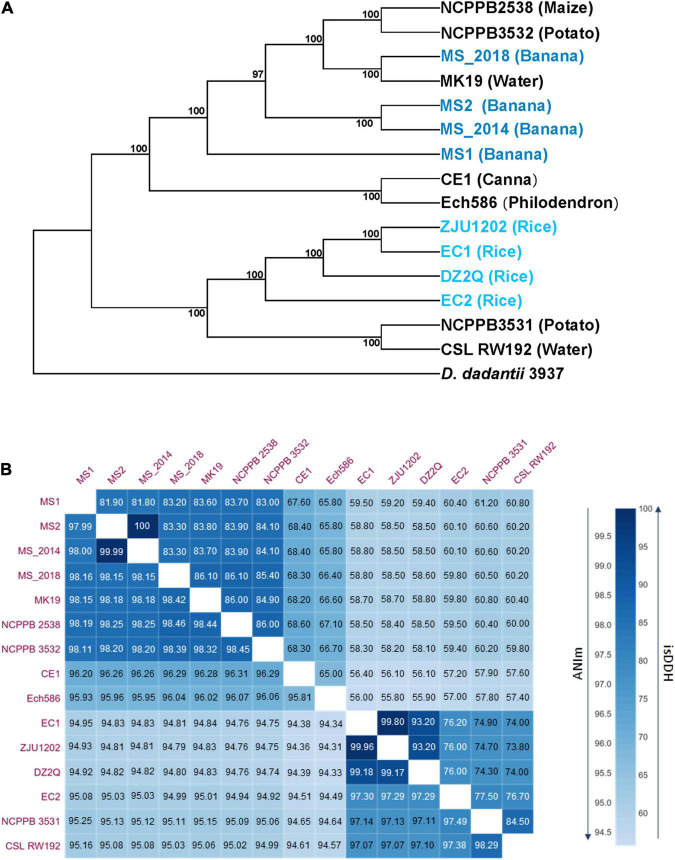
Phylogenetic, average nucleotide identity (ANI) and *in silico* DNA–DNA hybridization (isDDH) analyses of *D*. *zeae* strains based on genome sequences. **(A)** Phylogenetic analysis was conducted with the maximum likelihood method and bootstrapped at 1,000 replications, and *D*. *dadantii* 3937 was used as the out-group control. **(B)** Calculation of ANI and *is*DDH between pairs of genome sequences, upper numbers indicate *is*DDH values and lower numbers indicate the ANI values.

Pan- and core-genome analyses obtained a pan genome of 6,449 and a core-genome of 2,997 gene families among 15 analyzed *D*. *zeae* genomes and revealed an increase in the pan-genome size and decrease in the core-genome size with the addition of genomes (Supplementary Figure 1), indicating the genomic heterogeneity within this species. Using sequences of conserved genes, 15 *D*. *zeae* strains were clustered into two distinct subgroups: the banana-host and rice-host types (Figure 2A). Four strains isolated from banana showed obvious genomic divergence within the former subgroup. Unlike the strains from rice, strains from banana could not be considered within a closer clade representing strains from the same hosts. ANI and *is*DDH analyses supported these observations (Figure 2B). ANI values higher than 96% and *is*DDH values higher than 70% were found within subgroups rather than between subgroups. Strains NCPPB 3532, NCPPB 2538, and MK19 were close to those four strains from banana, and all ANI and *is*DDH values for each pair were higher than 96 and 70%, respectively. However, the four strains from banana did not have closer relationships, indicating significant genomic differentiation among these strains.

### Conserved Genomic Features of Strains From Banana in Most of the Virulence Determinants

Type I–VI secretion systems (T1SS–T6SS) were predicted for each of the four strains from banana (Supplementary Table 1), similar to other strains of *D*. *zeae*. The gene cluster *prtA*–*prtG* of T1SS, *outS*-*outO* of T2SS, and *hrpN*-*hrcU* of T3SS were homologous among the four strains from banana, indicating a highly conserved feature in these three secretion systems. In T5SS and T6SS, MS2, MS_2014, and MS_2018 had two T5SS loci and three T6SS loci. However, MS1 had only one T5SS locus and four T6SS loci. Further, *cdiA1*/*2* and *rhsA*/*B*/*C* at each homologous gene locus were found to be conserved at their N-termini and highly varied at their C-terminal toxin domains. Additionally, a complete set of T4SS gene clusters was found only in MS1. The other three strains had parts of them: MS2 and MS_2014 had *virB1*-*virB2*, and MS_2018 contained *virB1*, *2*, *4*, *7*, *9*, *10*, *11*, and *eexN* without *virB5*, *6*.

Most plant cell wall degrading enzymes (PCWDEs) were conserved among the four analyzed strains (Supplementary Table 2). They included genes encoding pectate lyase (*pel*), pectin lyase (*pnlG*), pectin methylesterase (*pem*), polygalacturonase (*peh*), pectin acetylesterases (*paeX* and *paeY*), oligogalacturonate lyase (*ogl*), rhamnogalacturonate lyase (*rhiE*), Kdg regulator genes, endoglucanase (*celY* and *celZ*), beta-glucosidase (*bgxA*, *bglA*, *bglB*, *nagZ*, and *celH*), alpha-glucosidase (*lfaA*), xylanases (*xynA*), and xylose degradation enzymes (*xylA*/*B*/*F*/*G*/*H*/*R*). However, *kdgM* and *xynA* were absent in MS_2018 and *feaD* was absent in MS_2014. The beta-glucosidase genes *bglC* and *bglD*, polygalacturonase gene *pehW*, pectin methyl esterase gene *pemB*, and pectin lyase gene *pnlH* were not found in any of the four strains. However, only MS1 harbored the complete *ganA*/*B*/*C*/*E*/*F*/*G*/*K*/*L*/*R* gene sets, and MS2, MS_2014 and MS_2018 contained only *ganK*/*L*/*R*, which was scattered at each genome rather than arranged in cluster. Unlike *D*. *zeae* strains from rice, all strains from banana harbored both exopolysaccharide (EPS) synthesis genes (*wza*-*gnd*) and capsular polysaccharide (CPS) synthesis gene clusters (*cpsA*-GT1) (Supplementary Table 1), and each gene was highly homologous among these strains.

### Two Different Types of Gene Cluster *fliA*-*fliC* Found Within Strains From Banana

**FIGURE 3:**
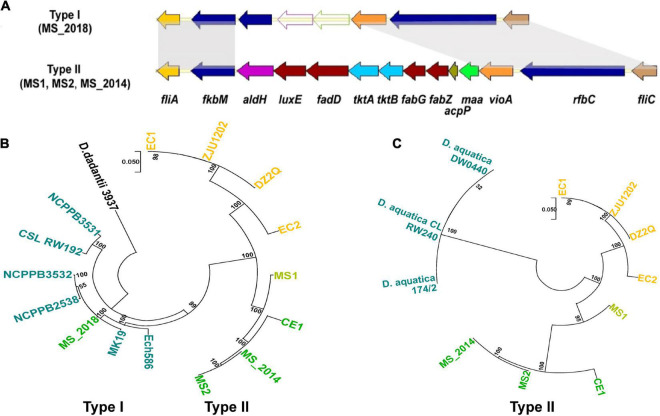
Genomic differentiation of *D*. *zeae* strains isolated from banana. **(A)** Genetic organization of *fli*/*che* clusters among *D*. *zeae* strains (genes aligned with a shadow are homologous). **(B)** Phylogenetic dendrogram based on *fliZ*-*flhD* genomic sequences of *D*. *zeae* strains with a maximum likelihood method and bootstrapped at 1,000 replications, *D*. *dadantii* 3937 was used as an out-group control. **(C)** Phylogenetic dendrogram based on *fkbM*-*rfbC* genomic sequences of *Dickeya* strains inserted with sugar biosynthesis and fatty acid biosynthesis gene clusters.

MS1, MS2, and MS_2014 had a complete flagellar biosynthesis and chemotaxis-associated gene cluster (*fli*/*che)* and included: the flagellin gene *fliC*, 38 flagellar biosynthesis genes, two flagellar motor genes and seven chemotaxis-associated genes. Similar to other *D*. *zeae* strains isolated in China, the locus of *fliA*-*fliC* was inserted by the sugar biosynthesis and fatty acid biosynthesis gene cluster *aldH*-*maa* (Figure 3A). Interestingly, the flagellar biosynthesis and chemotaxis-associated genes were conserved in MS_2018, but this strain contained another type of gene cluster (*fkbM*–*iolX*–*carA*) located within *fliA*-*fliC* which was named type I (Figure 3A). The *iolX and carA* genes encode oxidoreductase and carbamoyl-phosphate synthase, respectively. The former inserted gene cluster named type II was much different, where *maa* encoded maltose *O*-acetyltransferase, *tktA* and *tktB* encoded sugar biosynthetic proteins, and *aldH*, *luxE*, *fadD*, *fabG*, *fabG*, and *acpP* encoded fatty acid biosynthesis proteins. Phylogenetic analysis based on genomic sequences of *fliz*-*flhD* from *D*. *zeae* strains also showed distinct clustering into those two types (Figure 3B). Type I gene cluster was conserved in most *Dickeya* species, and gene clusters of MS_2018 and other foreign strains isolated from different hosts were grouped within this type. In another case, an inserted gene cluster was also found in a recently described species, *D*. *aquatica*, strains of which were isolated from freshwater rivers and still have no known host (Duprey et al., 2019). However, those inserted fragments in *D*. *aquatica* strains were highly conserved and could be distinguished from those in *D*. *zeae* strains, while *D*. *zeae* strains showed considerable differentiation at this locus (Figure 3C). Using inserted fragments, BLAST analyses revealed that MS1 had similar identities with strains from rice (EC1, ZJU1202: 89.50%; DZ2Q: 89.47; EC2: 89.17%), banana (MS2, MS_2014: 89.48%), and canna (CE1: 88.03%), while it shared lower identities with *D*. *aquatica* strains (76.51%). Notably, MS2 had higher identities with strains from banana (MS_2014: 100%; MS1: 89.48%) and from canna (CE1: 97.30%) than from rice (80.45-80.97%) and *D*. *aquatica* species (74.72%). Strains from rice also shared higher similarities between each other. In combination with the phylogenetic tree results (Figure 3C), MS1 could be considered an intermediate type between strains from banana and rice.

### Different Genomic Constituents of Secondary Metabolite Gene Clusters Within Strains From Banana and Related Phenotypic Variation

**FIGURE 4:**
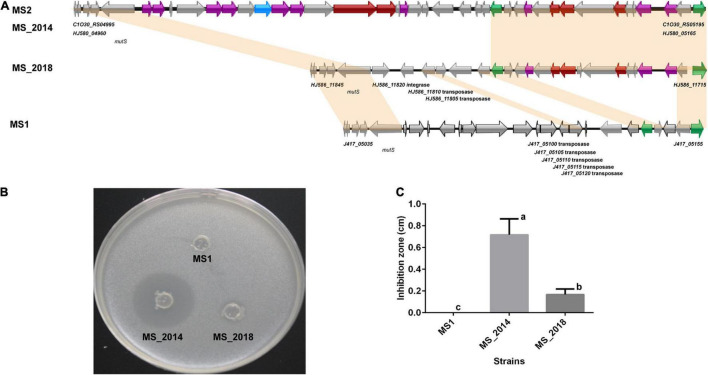
Comparison of the bacteriocin biosynthetic gene cluster and related phenotype among *D*. *zeae* strains isolated from banana. **(A)** Genomic organization of bacteriocin biosynthetic gene clusters (genes aligned with a shadow are homologous): 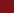 core biosynthetic genes, 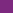 additional biosynthetic genes, 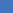 transport-related genes, 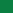 regulatory genes, 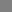 other genes. **(B)** Inhibition against the growth of *Escherichia coli* DH5α on LB agar after 24 h. The bacterial culture of strain MS_2014 was able to significantly inhibit the growth of *E*. *coli*, strain MS_2018 showed slight inhibition, and strain MS1 did not affect their growth. **(C)** Calculation of the inhibition zones on LB agar after 24 h. The inhibition zones were MS_2014 > MS_2018 > MS1, and they presented significant difference between each pairs and were labeled with different letters (a or b or c).

**FIGURE 5:**
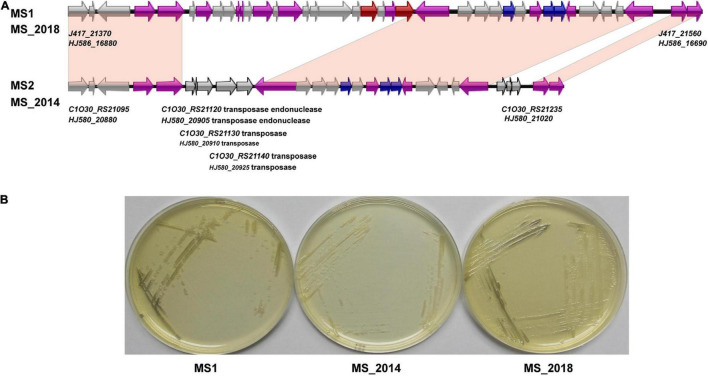
Comparison of the aryl polyene pigment biosynthetic gene cluster and related phenotypes among *D*. *zeae* strains isolated from banana. **(A)** Genomic organization of aryl polyene pigment biosynthetic gene clusters (genes aligned with a shadow are homologous): 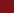 core biosynthetic genes, 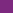 additional biosynthetic genes, 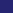 transport-related genes, 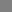 other genes. **(B)** Colony pigments produced after 48 h on LB agar. Strains MS1 and MS_2018 produced gray colonies compared to strain MS_2014.

AntiSMASH predicted eight types of secondary metabolite clusters among the four strains from banana and each strain contained seven of the clusters (Supplementary Table 3). Five of the clusters were predicted within all four strains: the hserlactone cluster, siderophore cluster, thiopeptide cluster (similar to the *O*-antigen saccharide biosynthetic gene cluster), transAT-PKs-NRPs cluster (similar to the cichopeptin biosynthetic gene cluster), and cyanobactins cluster. An NRP gene cluster similar to the turnerbactin synthetic gene cluster was found among the four strains and conserved in other *D*. *zeae* strains. However, the important core biosynthetic gene in MS_2018 (Locus *HJ586_02465*) was found to be frame shifted because of a neighboring inserted transposase gene (*HJ586_02470*). Another complete NRP gene cluster encoding ribosomally synthesized and post translationally modified peptide products (RiPP), such as bacteriocin, was found in MS2 and MS_2014 but not in MS1 or MS_2018. Strain MS_2014, harboring this bacteriocin cluster (Figure 4A), was able to inhibit the growth of *E*. *coli* DH5α, but MS1 and MS_2018 were not (Figures 4B,C). Compared to other *D*. *zeae* strains, only MS2 and MS_2014 had this larger gene cluster, while strains MS_2018, NCPPB_3532, NCPPB_2538, MK19, Ech586, and NCPPB 3531 were missing one of the core biosynthetic modules even though they were also predicted to have a bacteriocin gene cluster. However, a gene cluster encoding aryl polyene (APE) pigments was found in MS1 and MS_2018 but not in MS2 or MS_2014. Accordingly, colonies of strains MS1 and MS_2018 produced gray pigments in culture, but strain MS_2014, which was missing this APE cluster, did not (Figure 5).

### Variation of Direct Repeat Sequences and Clustered Regularly Interspaced Short Palindromic Repeats Loci Within Strains From Banana

**FIGURE 6:**
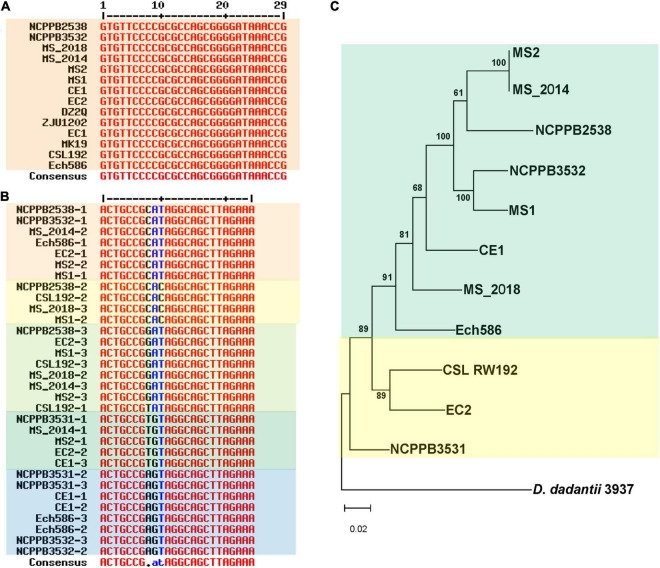
Genomic differentiation of *D*. *zeae* strains at clustered regularly interspaced short palindromic repeat (CRISPR) arrays. **(A)** Alignment of direct repeat (DR) sequences of type I-E CRISPR arrays. Strain NCPPB 3531 lost this array. **(B)** Alignment of DR sequences of type I–F CRISPR arrays. **(C)** Phylogenetic dendrogram based on genomic sequences of type I–F CRISPR arrays with the maximum likelihood method bootstrapped at 1,000 replications, *D*. *dadantii* 3937 was used as an out-group control.

Clustered regularly interspaced short palindromic repeat comprise direct repeat (DR) sequences and CRISPR-associated gene (Cas) sequences. Analyses of the genomes of *D*. *zeae* strains revealed three distinct types of CRISPR arrays: type I-F, type I-E, and type III-A. DR analogs (GTCTTCCCCACGCACGTGGGGGTGTTTCT) were highly conserved in the type I-E CRISPR array, except that NCPPB 3531 lost this array (Figure 6A). However, the type I-F CRISPR array varied among the different analyzed strains and at three base pairs of DR analogs (ACTGCCGNNNAGGCAGCTTAGAAA). However, the features of DR analogs were irregular among these analyzed strains, and strains from banana showed different types of DR sequences that were distributed within the analyzed strains (Figure 6B). Phylogenetic analysis based on genomic sequences of the type I–F CRISPR locus was similar to that based on whole genome sequences, indicating two distinct groups represented by strains from banana and rice (Figure 6C). Four strains from banana clearly varied at this locus; MS1 was closer to MS2 and MS_2014, while MS_2018 was closer to Ech586 and CE1. Additionally, only MS_2018, Ech586, and NCPPB 3531 contained a type III-A CRISPR array. DR analogs within these three strains were more diverse than other CRISPR arrays.

### Genomic Dissimilarities Related to Gene Islands

Whole-genome comparison using BLAST-Atlas analysis revealed that conserved genes were evenly distributed through each representative genome. Dissimilarities were also spread over entire genomes, as indicated by obvious genomic differentiation, except for the pair of MS2 and MS_2014. Comparing those major dissimilarities to predicted GIs, we found that most were matched to GIs, and genes encoding integrases, conjugative elements, transposases, and phage elements were abundant within these GIs (Supplementary Table 4).

**FIGURE 7:**
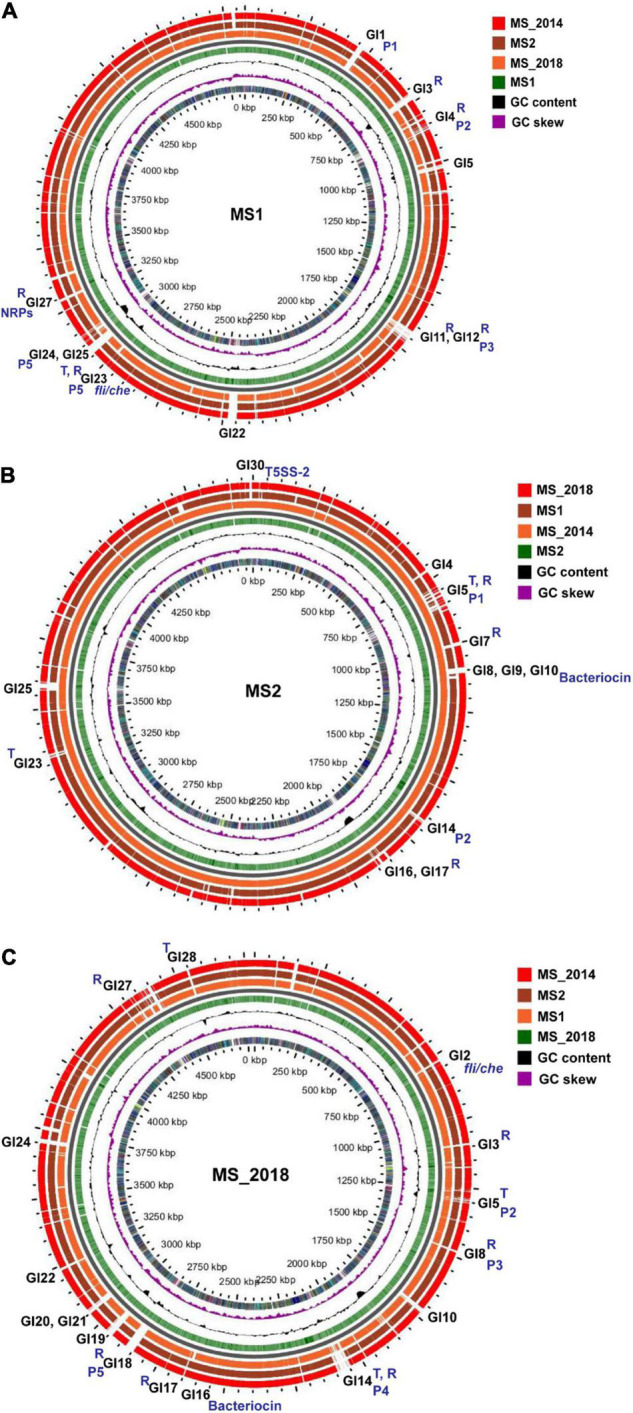
Genome BLAST atlas of *D*. *zeae* strains isolated from banana. **(A)** Comparison using MS1 as the reference genome. **(B)** Comparison using MS2 as the reference genome. **(C)** Comparison using MS_2018 as the reference genome. In each map of genome BLAST atlas, circles from outside in, –3, –2, and –1, indicate the query genomes, 0 indicates the backbone, 1 indicates the reference genomes, 2 indicates GC Contents on the reference sequence, 3 indicates GC Skew on the reference sequences, and 4 indicates COG categories of CDS regions on the reference sequences. Regions containing transposons and repeats are marked with T and R, respectively; regions corresponding to genomic islands and prophages are marked with GIs and Ps, respectively; regions corresponding to *fli*/*che*, bacteriocin, and NPRs are also labeled specifically.

Based on MS1, 11 GIs were matched to major dissimilar regions (Supplementary Table 4). At MS1-GI23, *fli*/*che* clusters presented an obvious dissimilarity between MS_2018 and other strains (Figure 7A), and an IS3 family transposase gene (*J417_13540*) was found next to *flhD*. Adjacent to MS1-GI23, an intact large prophage gene cluster was found in MS1-GI23-GI26 (Figure 7A). Within the MS1-GI27 neighboring the T2SS cluster, an NPR gene cluster (Locus *J417_14755-J417_14885*) similar to turnerbactin was present (Figure 7A) and contained several inserted continuous genes (*J417_14750-J417_14800*), compared to the other three strains (Supplementary Figure 2). *J417_14750* (Supplementary Figure 2), encoding an integrase, was somewhat similar to a virulence factor of Sai integrase, *intC*, in *Shigella flexneri*. The remaining genes of this gene cluster were mostly homologous among the four strains from banana (Supplementary Figure 2), and *J417_14830*, *J417_14845*, and *J417_14870* were predicted to be homologous with the virulence factors in VFDB, *fepD*, *entF*, and *entE*, respectively.

Based on MS2, 12 GIs were matched to major dissimilar regions. From MS2-GI8-GI10, a large bacteriocin gene cluster was found, as was conserved with MS_2014 (Figure 7B). Compared with MS1 and MS_2018, they harbored a genomic insertion at the bacteriocin gene clusters. Unlike MS1, fewer secondary metabolite clusters were found within the predicted GIs of MS2, and gene clusters similar to siderophore and turnerbactin could be found within MS1-GI13 and MS1-GI27, respectively, but not in the GIs of MS2 and MS_2014. Moreover, *fli*/*che* clusters were not matched to the predicted GIs of MS2 and MS_2014. However, we found that *cdiA2* (Locus *C1O30_RS20150*) and *cdiB2* (*C1O30_RS20160*) in T5SS-2 were included within MS2-GI30 (Figure 7B), as well as IS3 transposase genes (*C1O30_RS20075*). This was similar to MS_2014 (*cdiA2*: Locus *HJ580_19930*, *cdiB2*: *HJ580_19940*, IS3: *HJ580_19855)*, but MS1 did not have this *cdiA2*/*B2* locus. Additionally, MS2-GI28 consisted of genes (*C1O30_19120-C1O30_RS19150*) encoding several transcriptional regulators, one RelE/ParE family toxin, one endonuclease, and one integrase, as were also conserved in MS_2014. Interestingly, we found that these genes were inserted into a gene cluster encoding cyanobactin in MS2 and MS_2014 but not in MS1 and MS_2018, while the remaining genes were all conserved among the four strains from banana.

Based on MS_2018, 15 GIs were matched to major dissimilar regions. Another type of *fli*/*che* cluster was located at MS_2018-GI2 (Figure 7C), and there was no IS3 gene next to *flhD*. At MS_2018-GI16, an incomplete bacteriocin gene cluster (*HJ586_11720-HJ586_11770*) was found. Next to this locus, *HJ586_11805-HJ586_11820* was located at MS_2018-GI17, most of which encoded transposases and integrases and were not conserved in other strains from banana. Similar to MS2 and MS_2014, MS_2018-GI25 also contained *cdiA2* (*HJ586_17825*) and *cdiB2* (*HJ586_17815*), except that the IS3 genes were not in the same neighborhood.

### Prophage Elements Presenting the Relationship of Strains From Banana

**FIGURE 8:**
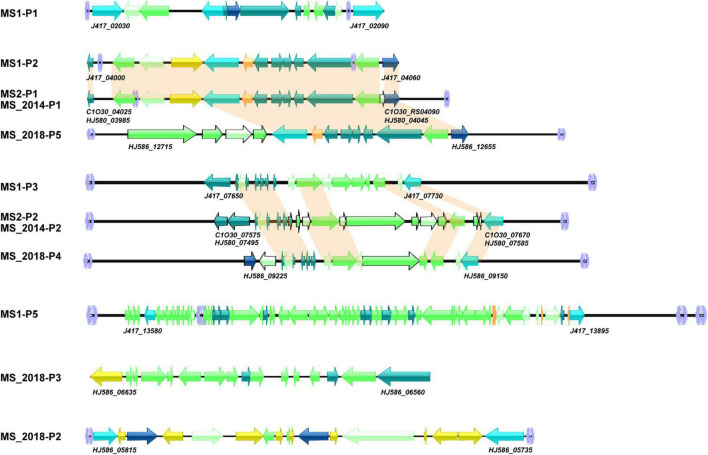
Genomic organization of prophage gene clusters of *D*. *zeae* strains isolated from banana. Arrow position represents forward/reverse gene orientation (genes aligned with a shadow are homologous). Arrow colors indicate specific gene composition, 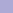 *att* loci, 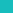 integrases, 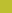 transposases, 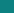 phage-like proteins, 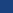 tail shaft proteins, 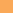 tRNA, 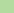 other genes, 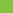 hypothetical proteins.

Phages and prophages are important mobile elements in bacterial genomes. MS1 had a large intact prophage at MS1-GI23-GI26 that was well discriminated from the other three strains from banana. Thus, we searched their prophage elements and compared them with those of other *D*. *zeae* strains. Five, three, three, and five prophages were detected, respectively, in MS1, MS2, MS_2014, and MS_2018; MS2 and MS_2014 shared common prophage loci and were conserved at most constituent genes. More interestingly, all three prophages detected in MS2 and MS_2014 had allelic prophages with the MS1 genome (Figure 8). In MS1, prophages MS1-P1 (*J417_02030-J417_02085*), MS1-P2 (J*417_04000-J417_04060*), MS1-P3 (*J417_07650-J417_07730*), and MS1-P5 (*J417_13580-J417_13895*) were found at MS1-GI1, MS1-GI4, MS1-GI11, and MS1-GI23-GI26, respectively. Another prophage, MS1-P4, contained genes highly conserved among *D*. *zeae* strains and was found in genomically similar regions rather than GIs (Supplementary Table 4). Both strains MS2 and MS_2014 had prophages P1 (MS2-GI5, MS_2014-GI5), P2 (MS2-GI15, MS_2014-GI14-15), and P3 (not located within GIs) that were allelic to MS1-P2, MS1-P3, and MS1-P4, respectively. P1 and P2 in MS2/MS_2014 showed differences in some genes with P2 and P3 in MS1 (Figure 8). Compared with MS_2018 and other *D*. *zeae* strains, MS1-P2, MS2-P1, and MS_2014-P1 had closer relationships at those allelic prophages (Figure 8). In MS_2018, P2, P3, P4, and P5 were detected within GI5, GI8, GI14, and GI18, respectively (Supplementary Table 4). P1 was conserved with the other analyzed strains and not found in GIs; P2 and P3 were specific in MS_208. Likewise, MS_2018-P4 and MS_2018-P5 differed from other strains isolated from banana (Figure 8).

### Diversity of Virulence Among Different Banana Varieties

**FIGURE 9:**
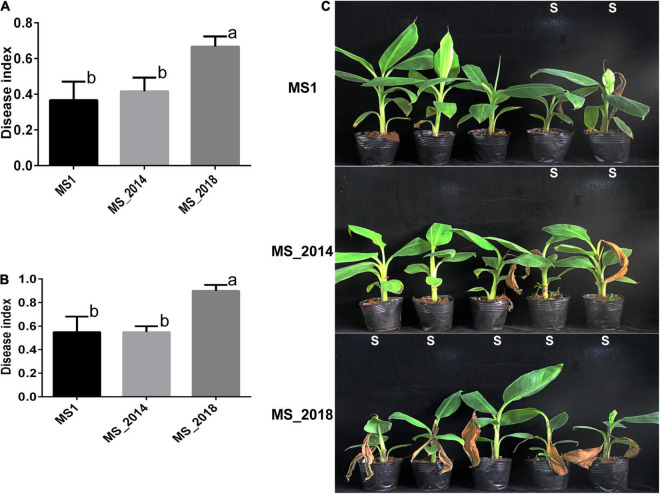
Development of soft rot symptoms after inoculation with *D*. *zeae* strains isolated from banana. **(A)** Calculation of disease indexes of banana seedlings of *Musa* AAA Cavendish 7 days after inoculation. **(B)** Calculation of disease indexes of banana seedlings of *Musa* ABB Plantain 7 days after inoculation. Significant difference was indicated by different letters (a or b or c), while the labels with the same letter indicates no significant difference. **(C)** Symptoms of banana seedlings of *Musa* AAA Cavendish 7 days after inoculation, S indicates seedling is susceptible to pathogen and shows soft rot symptoms.

All four varieties of *Musa* ABB Pisang Awak (Guangfen-1, Qingfen, Pinguofen, and Fenza) were sensitive to the three strains isolated from banana, and with no obvious difference in virulence among the tested strains. Varieties of Cavendish (*Musa* AAA) and Plantain (*Musa* ABB) showed considerable resistance against strains MS1 and MS_2014, and most inoculated bananas exhibited typical soft rot symptoms at the newly emerging leaves and slight symptoms of the pseudostem (Figure 9 and Supplementary Table 5). However, MS_2018 was more virulent to these two banana varieties and caused more severe soft rot symptoms on both newly emerging leaves and pseudostems, and on more inoculated plants (Figure 9). Using slides of potato tubers, the areas of disease spot were larger on slides inoculated with MS_2018 than on slides inoculated with MS1 or MS_2014 and there was a significant difference between MS_2018 and MS1, indicating a stronger ability of the pathogenic bacteria to induce soft rot symptoms (Supplementary Figure 3).

## Discussion

### *Dickeya zeae* Strains From Banana Might Have Diverged Earlier From Ancestral Strains Than Strains From Other Hosts in China

In Guangdong Province, China, bacterial soft rot of banana caused by *D*. *zeae* was first described in 2009 by our group (Lin et al., 2010). *Dickeya zeae* causing foot rot of rice, however, was first reported several years before (Hussain et al., 2008). Investigation of ANI and *is*DDH pairwise values (Figure 2) revealed that Ech586 and CE1 fell between subgroups of the banana and rice-host types, as well as another strain, JZL7, isolated from Clivia in Guangdong, China (Hu et al., 2018). Based on concatenated sequences of housekeeping genes, a phylogenetic dendrogram (Supplementary Figure 4) presented a similar evolutionary relationship among the analyzed bacterial strains. In comparison with strains isolated from rice (*Pi*, 0.00830), the strains isolated from banana had a higher nucleotide diversity (*Pi*, 0.01120), indicating that they should have diverged sooner from ancestral *D*. *zeae* strains than those from rice. Likewise, phylogenetic placement of the strains Ech586, CE1, and JZL7 also fell between strains from banana and rice. These observations were indicated by the specific genomic features of fatty acid and sugar biosynthesis genes inserted within the *fliZ-flhD* locus. The strains isolated from banana had higher nucleotide diversity (*Pi*, 0.07141) in the sequences of fatty acid and sugar biosynthesis genes than the other two types of strains (rice: *Pi*, 0.02033; canna and Clivia: *Pi*, 0.00681). Of more interest, the *F*_ST_ value between strains from banana and strains from canna and Clivia was 0.09554, indicating a frequent gene flow. Thus, in China, strains isolated from banana might have diverged earlier from ancestral strains than strains from other hosts. Bacterial strains diverged from foreign or local ancestral strains to adapt and infect banana, and then evolved to adapt new hosts by exchanging useful genomic fragments with other bacteria. The *D*. *zeae* pathogen may have been infecting banana for many years, but the disease “bacterial soft rot of banana” was not established until 2009. The soft rot was erroneously identified previously as *Fusarium* wilt because of similar symptoms on the leaves and basal pseudostem. With changes in cropping patterns and cultivated banana varieties, the bacterial disease outbreak that occurred in Guangzhou, Guangdong, China, in 2009 has since then attracted more attention. That soft rot bacteria are evolving and adapting to new hosts, such as canna and Clivia, also highlights the economic importance of bacterial soft rot diseases on economically important crops.

### Differentiated Virulence Determinants Reside Mainly in Genomic Islands Abundant With Integrases and Transposases

T1SS–T6SS are key for the pathogenesis of *Dickeya* since they transport virulence components (Charkowski et al., 2012), and T1SS and T2SS are responsible for translocation and secretion of PCWDEs, including pectinases, polygalacturonases, cellulases, and proteases (Lindeberg et al., 1998; Alvarez-Martinez and Christie, 2009). These virulence components were mostly conserved among the strains from banana. A periplasmic endogalactanase gene cluster, *ganA*/*B*/*C*/*E*/*F*/*G*, was responsible for the degradation of galactan chains in pectin-ramified regions (Hugouvieux-Cotte-Pattat et al., 2014) and was found to be conserved in most previously reported *D*. *zeae* strains, including MS1. The *gan* gene cluster was likely present in the ancestor of *Dickeya* spp. (Duprey et al., 2019), and is considered secondarily lost in some strains from banana, as well as and phylogenetically close strains like NCPPB 2538, NCPPB 3532, and MK19. These virulence factors were mainly distributed in genomically matched regions (Figure 7). Thus, we paid more attention to large genomically unmatched regions.

Notably, most of these genomic dissimilarities resided in their predicted GIs. From MS1-GI23 and MS_2018-GI2, we found the differentiated *fli*/*che* clusters. Flagellar biosynthesis and chemotaxis proteins are associated with virulence in *D*. *dadantii* pathogens (Jahn et al., 2008; Antúnez-Lamas et al., 2009), so the relationship of this genomic differentiation and virulence variation should be considered in the future. The *fli*/*che* cluster, however, was not present within the predicted GIs of MS2 and MS_2014. In other hand, the two-partner secretion system, T5SS functions in the pattern of contact-dependent growth inhibition by delivering the C-terminal toxin domain of CdiA (Pédron et al., 2014), and two T5SS loci were found among *D*. *zeae* strains. T5SS-1 was conserved, while T5SS-2 showed genetic differentiation among strains from banana and also found within GIs MS2-GI30, MS_2014-GI30, and MS_2018-GI25.

Secondary metabolites with the bioactivities of antibiosis, anticancer agents, and many other drugs are supposed to play important roles in environmental adaptation and microorganism competition or to act as virulence factors (Minowa et al., 2007). NRPs and PKs are enzymes responsible for the synthesis of these secondary metabolites, and they are recruited into highly modular compact gene clusters containing pathway-specific regulatory and transport genes (Medema et al., 2011). Strains from banana commonly contained most genomic loci that were involved in the biosynthesis of secondary metabolites, their genomic differentiation could result in different bacterial phenotypes and was usually present within the predicted GIs.

First, the varied bacteriocin gene cluster and was in the GIs of all four strains: MS1-GI7, MS2-GI8–GI10, MS_2014-GI8–GI10, and MS_2018-GI16–17. In the first core biosynthetic module, MS1 and MS_2018 were rich in integrase and transposase genes (Figure 4A). The DNA mismatch repair gene *mut* was next to these two types of genes and conserved among different *D*. *zeae* strains. This might indicate a hot spot of genomic rearrangement.

Second, the APE pigment gene cluster was found within MS2-GI32 since the IS3 transposase genes replaced the core biosynthetic genes and several additional biosynthetic genes (Figure 5A). Therefore, the remaining genes in MS2 and MS_2014 were unable to produce APE pigments. More interestingly, these core and additional biosynthetic genes were also lost in most strains of the rice-host type, including EC1, EC2, ZJU1202, DZ2Q, and CSL RW192. Therefore, strains such as EC1 did not produce gray pigments (Zhang et al., 2014), as well as EC2 (data not shown). However, those IS3 genes were not found in the corresponding loci of these strains. APE pigment gene clusters were conserved among most *D*. *zeae* strains, including newly reported strains JZL7, PL65, and A5410, and should have existed in the ancestors of *D*. *zeae* strains. This evidence may suggest that strains isolated from rice diverged more recently and lost the IS3 genes, causing their inability to produce APE pigments.

Third, most genes of the NPR gene cluster encoding turnerbactin were conserved among *D*. *zeae* strains from different hosts, but they were also found to be present in MS1-GI27 and MS_2018-GI1. In MS1, an integrase (*J417_14750*), followed by several other genes, was not conserved but only present in CE1 with some difference, while the core biosynthetic gene of NPRs (*entF*) was truncated by a transposase in MS_2018. The integrases and transposases increased the genomic differentiation within the GI regions, and their abundance within GIs indicated they could have played important roles in the acquisition and loss of essential genes during genomic differentiation.

### CRISPR Constituents and Prophage Elements Greatly Contributed to Bacterial Differentiation

To fully illustrate the evolutionary relationships among strains isolated from banana, genomic diversity of extrachromosomal origin, such as CRISPRs and prophages, was considered in this study. CRISPRs protect against bacteriophages or plasmids by direct sequence-specific destruction of their DNA or other genetic material (Horvath and Barrangou, 2010). Unlike type I-E and type I-F CRISPR arrays, type III-A CRISPR arrays were present in minor strains. They were found in both banana-host type (MS_2018, Ech586, and PL65) and rice-host type (NCPPB 3531) strains. However, this recently acquired CRISPR array varied significantly among those holding strains. Their DR sequences obviously varied in length and base constituents. Generally, DR sequences are highly conserved within the CRISPR locus (Deveau et al., 2010). Thus, the variations occurring in the DR sequences indicate that this genomic constituent might be under selection pressure and have been undergoing degeneration of repeat sequences (Kunin et al., 2007). Furthermore, the genomic organization of their *cas* gene displayed a genomic rearrangement at *cas1* and *cas2* (Supplementary Figure 5). In this genomic feature, MS_2018 was closer to Ech586, while PL65 was more conserved with NCPPB 3531, possibly indicating a frequent exchange between banana and rice-host type strains. Type III-A CRISPR-Cas systems in *Staphylococcus aureus* and *S*. *epidermidis* have nonspecific DNase activity and increase mutations in host pathogens. Thus, they can modulate the evolution of bacteria by generating genetic diversity (Mo et al., 2021). Whether the later acquired type III-A CRISPR system modulated the evolution of those carrier strains of *D*. *zeae* in their adaptation to plants or the environment is a question worth answering in future studies.

Prophage elements are intracellular forms of bacteriophages that are integrated ubiquitously into bacterial genomes (Casjens, 2003). They play important roles in virulence (Dominguez-Mirazo et al., 2019), biofilm formation (Shen et al., 2018), and host immunity (Gentile et al., 2019). In this study, we found intact and incomplete prophages in the strains from banana, and most located in their predicted GIs except that one prophage was conserved among them and not located in GIs. Specific genes of strains from banana, compared with other *D*. *zeae* strains (Supplementary Table 6), were grouped into two major genomic regions that were also matched to GIs. More interestingly, we found that both specific regions were adjacent to the prophage elements. The first specific regions in MS1-GI4 (*J417_03660* –*J417_04000*), MS2-GI5 (*C1O30_RS03600*–*C1O30_RS04025*), and MS2014-GI5 (*HJ580_03565*–*HJ580_03985*) were exactly adjacent to MS1-P2 (*J417_04000*–*J417_04060*), MS2-P1 (*C1O30_RS04025*–*C1O30_RS04090*), and MS2014-P1 (*HJ580_03985*–*HJ580_04045*), respectively. The second specific regions in MS1-GI26 (*J417_13900*–*J417_13915*) were exactly adjacent to MS1-P5 (*J417_13580*–*J417_13895*). However, these specific genes in MS_2018 were not located within their predicted prophage regions. Thus, the relationship between specific genes and prophage elements might indicate an important role of prophages in bacterial evolution, as well as the evolving relationship of those strains from banana (MS1, MS2, MS_2014, and MS_2018). In the genomic features of prophages, MS2 and MS_2014 were closer to MS1, since all three prophage regions found in the former were found in allelic genomic regions of MS1 rather than in MS_2018 (Figure 8). MS_2018-P5 was not located in the allelic region of the other three strains from banana and this strain had two other specific prophage regions, indicating genomic differentiation. Near to *fli-che* cluster, MS1 had a large intact prophage, MS1-P5, that was not conserved in strains MS2, MS_2014, and MS_2018. This intact prophage was found in some *D*. *zeae* strains, such as MK19, EC1, NCPPB 3531, and CSL RW192, indicating that it might have been present in the ancestor of this species.

Clustered regularly interspaced short palindromic repeat constituents and prophage elements were evident in the genomic dissimilarities of different strains from banana, and contributed to their genomic differentiation. Considering the properties of CRISPR constituents and prophage elements, MS2 and MS_2014 were closer to MS1, while MS_2018 was more similar to foreign strains. Genomic features that might have been present in *D*. *zeae* ancestors, such as prophage P1, the APE pigment gene cluster, and *gan* cluster (Duprey et al., 2019), were all found in MS1, while MS2 and MS_2014 had lost them. However, the latter recently acquired the enhanced bacteriocin gene cluster, indicating that MS2 and MS_2014 might have diverged later than MS1. Additionally, a transposase gene found next to the *flhD* gene of MS1 suggested that this strain had earlier acquired the inserted fatty acid and sugar genes in the *fli-che* locus, which was supported by the phylogenetic analysis of inserted sequences (Figure 3C).

### Phenotypic Diversity Induced by Genomic Differentiation Brings Attention to Disease Control

The strains were isolated from banana in different years, but all strains in Nansha, Guangzhou, China showed obvious genomic differentiations and differed in pathogenicity and cultural characteristics. The most important phenotypic variation was in the pathogenicity on different banana varieties. In our previous studies, we first found bacterial soft rot of banana on a variety of *Musa* ABB Pisang Awak in Nansha, Guangzhou (Lin et al., 2010), and the varieties of *Musa* ABB Pisang Awak were more sensitive to soft rot pathogens than varieties of *Musa* AAA Cavendish and *Musa* ABB Plantain (Pu et al., 2012). Consistent with genomic differentiation and the possible evolving relationship occurring between MS_2018 and other strains isolated from banana, MS_2018 had a greater pathogenicity on the varieties previously considered moderately resistant or moderately susceptible. The emergence of genomically diverged strains in China, such as MS_2018, might be due to foreign entry of bacterial strain or changes in the dominant bacterial population allowing adaptation to various host varieties. Thus, we should pay more attention to the enhanced pathogenicity among other banana varieties, which might raise the risk of the spread of new variants of bacterial soft rot pathogens and the damage of this bacterial disease in banana growing regions.

Bacteriocins are RiPP-like natural product secreted by a variety of bacteria to kill other bacteria (Hetrick and van der Donk, 2017; Benítez-Chao et al., 2021). Strains from banana showed significant diversity in this antibiosis phenotype because of different core components in their biosynthetic gene cluster. MS_2018 had genes similar to those encoding RiPP-like thiostreptamide (Eyles et al., 2020); MS2 and MS_2014 contained one more specific NRP protein (*C1O30_RS05075*, *HJ580_05040*) with a domain similar to that of *Streptoalloteichus tallysomycin* biosynthesis genes; no such genes or proteins existed in MS1. This differentiation was according to their different abilities to inhibit bacterial growth. Bacteriocins help bacteria compete with other microorganisms (Cotter et al., 2013), and varied antibiosis phenotypes of bacteria might regulate their interaction with host plants and the microbial environment. Another important variation in phenotype was in APE pigments, these bacterial polyketides distributed widely in Proteobacteria and Bacteroidetes and often found in host-associated bacteria (Cimermancic et al., 2014). In this study, strains from banana showed different phenotypes in colony pigments related to the APE pigment cluster. There are no reports of APE pigments in *Dickeya* or *Pectobacterium*, but the APE pigments, including carotenoids, xanthomonadin, arcuflavin, and flexirubin have been identified (Schöner et al., 2016). They reportedly protect against oxidative stress in a variety of different bacteria, including *Escherichia*, *Variovorax*, *Lysobacter*, *Xenorhabdus*, and *Xanthomonas* (Grammbitter et al., 2019; Johnston et al., 2021) and also participate in biofilm formation (Johnston et al., 2021). APE pigments also help bacteria adjust their interactions with the surrounding environment and host immune systems (Johnston et al., 2021). Therefore, the presence or loss of the ability to produce APE pigments might depend on their surrounding factors, like banana varieties and crop environments. Together, the competition with environmental microorganisms and adaption to different environments might contribute to successful colonization and infection among various banana soft rot pathogens. These give a warning of the effectiveness of the strategies like using microbial control or adjusting bacterial fitness, and can aid disease control efforts by altering practicable strategies.

Heterogeneity in genomic content and virulence factors of pectinolytic bacteria are challenging for materializing strategies of developing resistant germplasm. The isolation and collection of different bacterial variants could be used for screening resistant banana germplasm and varieties, and can contribute to develop effective soft rot disease management strategies. In the future, we will explore the mechanism leading to variation in pathogenicity and host adaptation, and further clarify the role of genomic diversity in virulence mechanism.

## Conclusion

To understand the roles of genomic differentiation that help *D*. *zeae* strains adapt to their hosts and cause important bacterial disease of banana, we performed genomic and the related phenotypic analyses. Phylogenetic tree analysis and genome-genome nucleotide comparison indicated obvious genomic diversion among the strains. The strains from banana were conserved in most analyzed virulence determinants, but we found some genomic dissimilarity in the flagellar gene cluster and several secondary metabolite gene clusters in the predicted GIs. Elements of extrachromosomal origin like CRISPRs and prophages also appear to found influence bacterial divergence and evolution. We discovered phenotypic diversity in antibiosis effects, colony pigments, and especially virulence among different banana varieties.

## Data Availability Statement

The datasets presented in this study can be found in online repositories. The names of the repository/repositories and accession number(s) can be found below: https://www.ncbi.nlm.nih.gov/genbank/, CP053012, CP053013, and CP053014 www.ncbi.nlm.nih.gov/, BioProject accession nos. PRJNA194072, PRJNA628811, and PRJNA628824; Sample accession nos. SAMN01991085, SAMN14751396, and SAMN14751832.

## Author Contributions

JZ, MA, and QY: conceived and designed the study. HS and XP: bacteria isolation, cultivation, and pathogenicity tests. DS: BIOLOG assays. JZ, DS, and XP: genome sequencing and assembly. JZ, MA, and BL: comparative analyzes. JZ and QY: first draft of the manuscript. MA and JH: manuscript revision. All authors read and approved the final manuscript.

## Conflict of Interest

The authors declare that the research was conducted in the absence of any commercial or financial relationships that could be construed as a potential conflict of interest.

## Publisher’s Note

All claims expressed in this article are solely those of the authors and do not necessarily represent those of their affiliated organizations, or those of the publisher, the editors and the reviewers. Any product that may be evaluated in this article, or claim that may be made by its manufacturer, is not guaranteed or endorsed by the publisher.

## Footnotes

1
www.fao.org/faostat/en/

2
www.displayr.com/

3
https://server.gview.ca/
