# Supplementary figures and images for: Genomic Comparisons and Phenotypic Diversity of Dickeya zeae Strains Causing Bacterial Soft Rot of Banana in China
Source: Front Plant Sci. 2022 Feb 9;13:822829. doi: 10.3389/fpls.2022.822829 (PMC8864124; doi:10.3389/fpls.2022.822829)

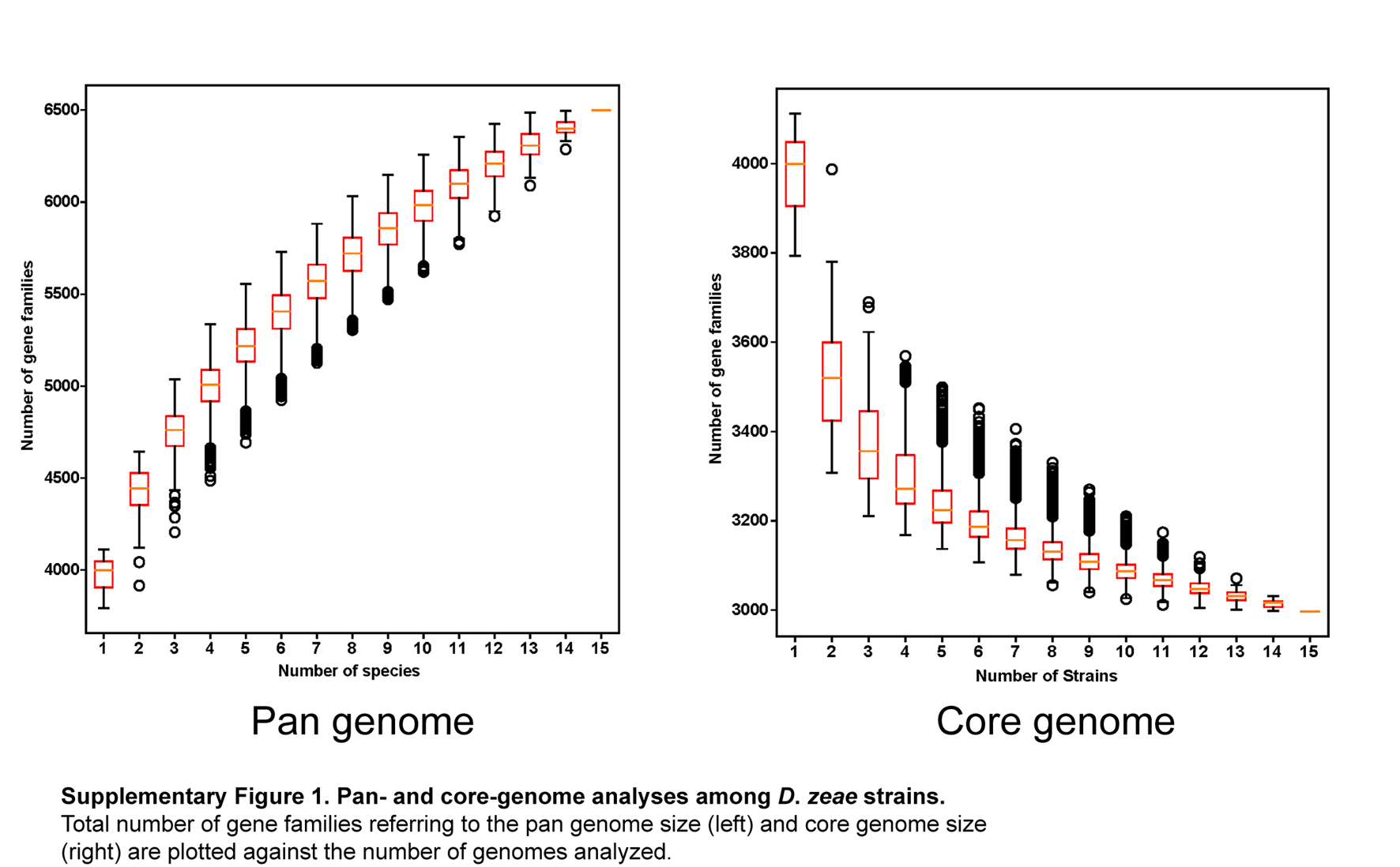

Supplement: Supplementary file 1 [file Image_1.TIF]

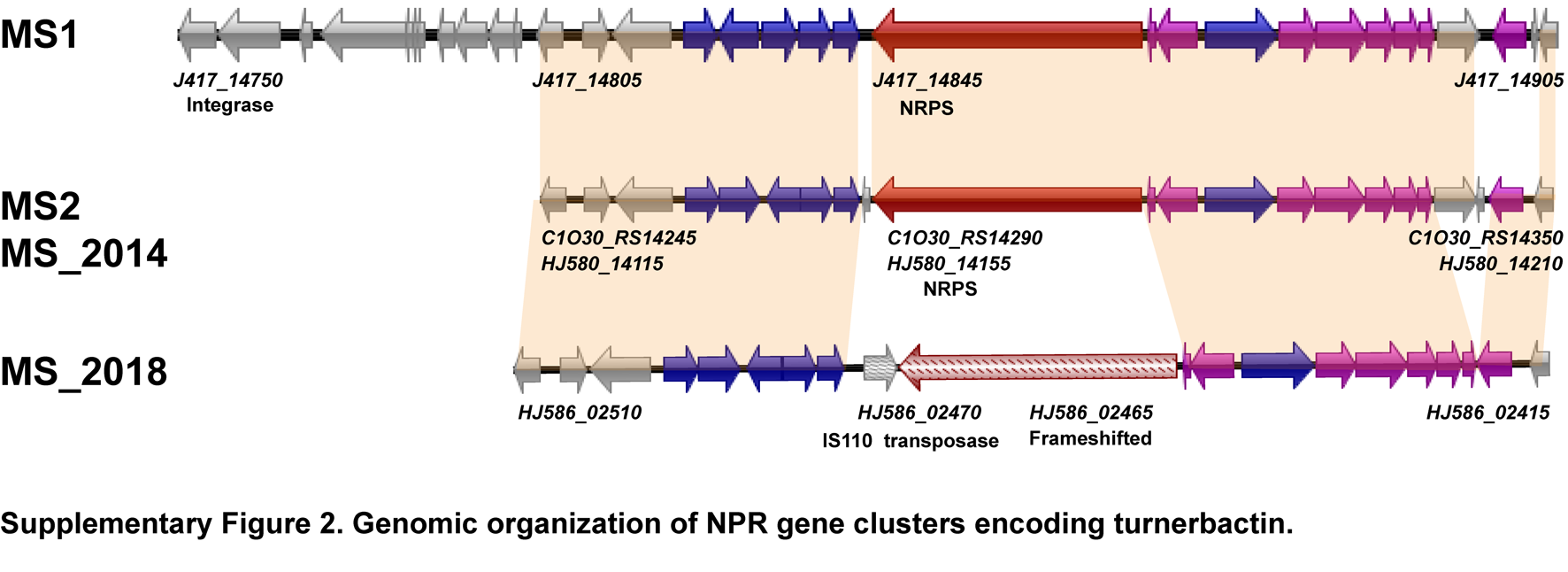

Supplement: Supplementary file 2 [file Image_2.TIF]

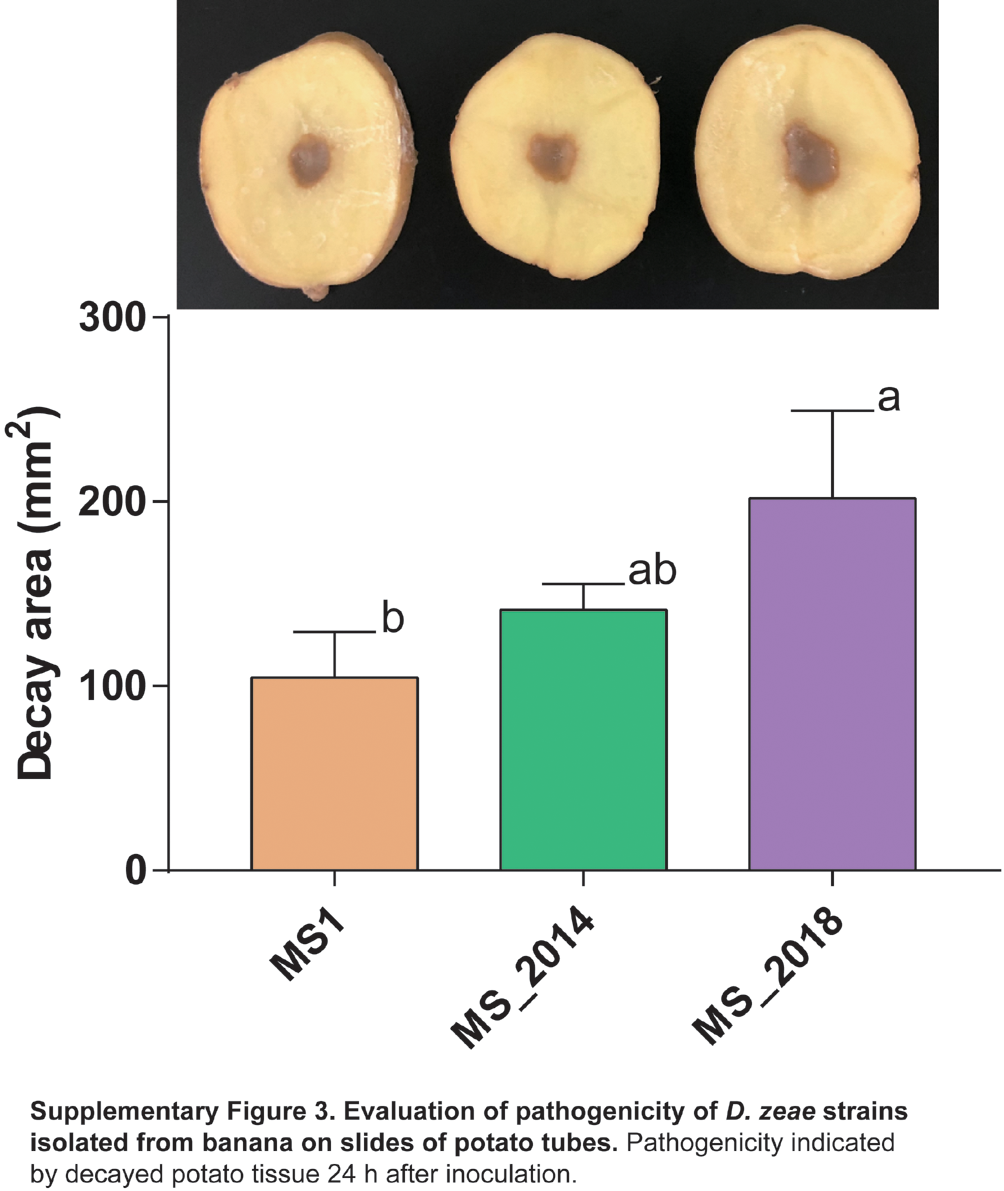

Supplement: Supplementary file 3 [file Image_3.TIF]

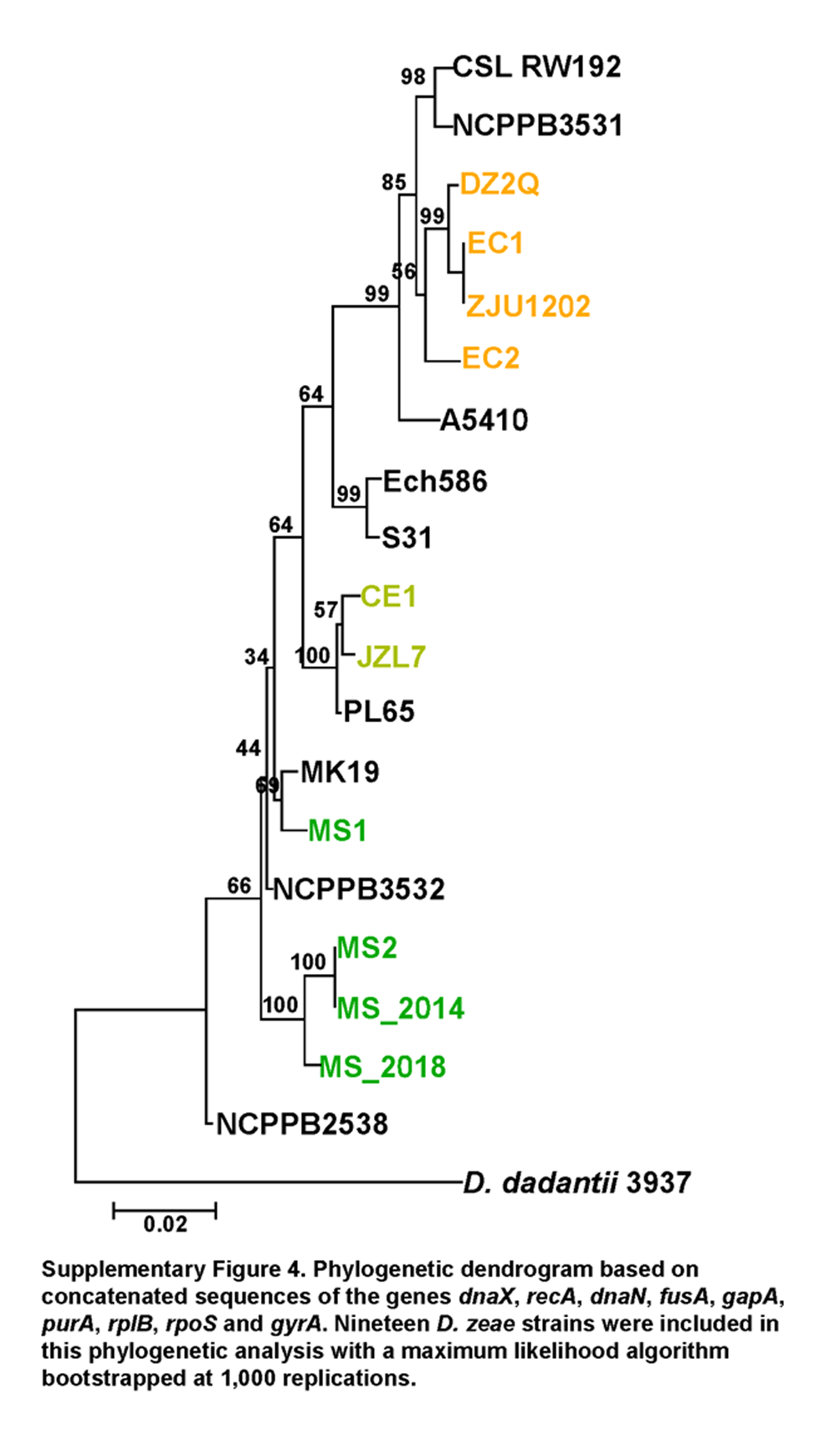

Supplement: Supplementary file 4 [file Image_4.TIFF]

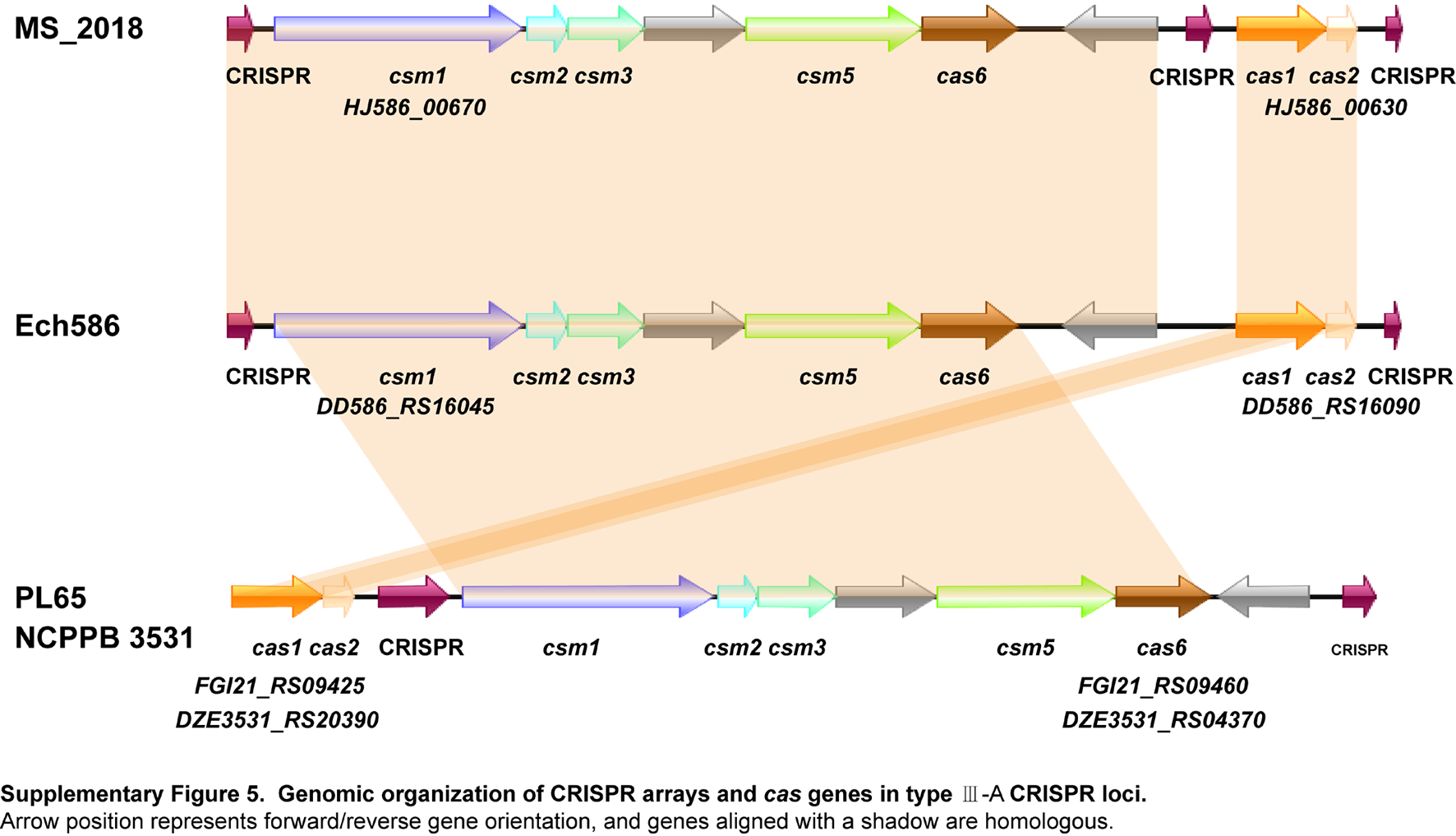

Supplement: Supplementary file 5 [file Image_5.TIF]
